# Two-center clinical validation of free breathing respiratory triggered retrospectively cardiac gated steady-state free precession (RT-SSFP) cine imaging in adults

**DOI:** 10.1186/1532-429X-18-S1-P263

**Published:** 2016-01-27

**Authors:** Amol Pednekar, Benjamin Cheong, Hui Wang, Scott Flamm, Raja Muthupillai

**Affiliations:** 1 Philips Healthcare, Houston, TX USA; 2 Radiology, CHI St. Luke's Health, Houston, TX USA; 3 grid.239578.20000000106754725Imaging Institute, Cleveland Clinic, Cleveland, OH USA

## Background

Notwithstanding all the advantages of the cine bSSFP there are certain limitations, especially in sedated patients, patients with impaired breath-holding capacity, and those with cardiac arrhythmias. Any respiratory motion due to unsteady breath-hold during data acquisition causes motion artifacts, sacrificing endocardial border definition. In this study we clinically validate a RT-SSFP sequence for free breathing acquisition of cardiac cine images.

## Methods

All imaging for this prospective, IRB approved study, was performed on 1.5T/3T commercial MR scanners (Achieva/Ingenia, Philips Healthcare) at two independent institutes (IA,IB). A real-time adaptive RT-SSFP sequence synchronizing the drive to SS with respiratory cycle along with retrospective cardiac gating was implemented[1]. The performance of RT-SSFP was validated prospectively against breath-hold (BH-SSFP) acquisition with identical acquisition parameters (TR/TE/flip angle = 2.5-2.7 ms/1.25-1.35 ms/ 70°; acqd voxel size = 1.7-2.0 × 1.6-2.0 × 8 mm^3^; SENSE factor = 1.3-1.9; temp res 40-50 ms; imaging time : 6-8 RR intervals/slice) for LV function evaluation in 22(IA,50(19-82)yrs) and 21(IB,47(23-86)yrs) consecutive patients undergoing clinically indicated cardiac MR. The image quality was scored by blinded reviewer for blood to myocardial contrast, endocardial edge definition, and motion artifacts (Table 1). Bland-Altman analysis was performed on clinical scores assigned to both the techniques.

**Table 1:** Clinical Score criteria

Score	BMC	Edef	Art
5	Excellent: Uniformly hyper intense blood pool with excellent contrast against myocardium; Myocardium is uniformly bright throughout the cardiac cycle with little evidence of flashing	Excellent: Papillary and endocardial trabeculae are clearly visible in the bright backdrop of blood pool	Excellent: Nearly artifact free images either due to motion or other factors such as banding, or localized signal losses
4	Good: Blood pool significantly brighter than myocardium or Myocardial signal intensity is fairly uniform throughout the cardiac cycle	Good: Papillary and endocardial trabeculae are visible but somewhat blurred during the cardiac cycle	Good: Some motion artifact is present, but does not affect overall image quality; Little or no other artifacts such as banding or localized signal losses
3	Moderate: Diagnostic quality images but with significant loss of blood to myocardial contrast or noticeable variation in myocardial signal through the cardiac cycle but does not hinder diagnosis	Moderate: Myocardial walls are clearly distinguished with barely distinguishable endocardial trabeculae	Moderate: Motion artifacts are visible but does not hinder diagnostic ability
2	Poor: Poor myocardial to blood contrast but still diagnostic	Poor: Both myocardial walls as well as endocardial trabeculae are significantly blurred	Poor: Nearly non-diagnostic images with significant artifacts
1	Non-diagnostic: Blood to myocardial contrast is poor and the images were deemed non-diagnostic	Non-diagnostic: Blood to myocardial edge definition is poor and the images were deemed non-diagnostic	Non-diagnostic image quality

BMC = Blood to myocardial contrast; Edef = end-diastolic volume; Art = ejection fraction; Combined = average (BMC, Edef, Art).

## Results

**Figure 1:**
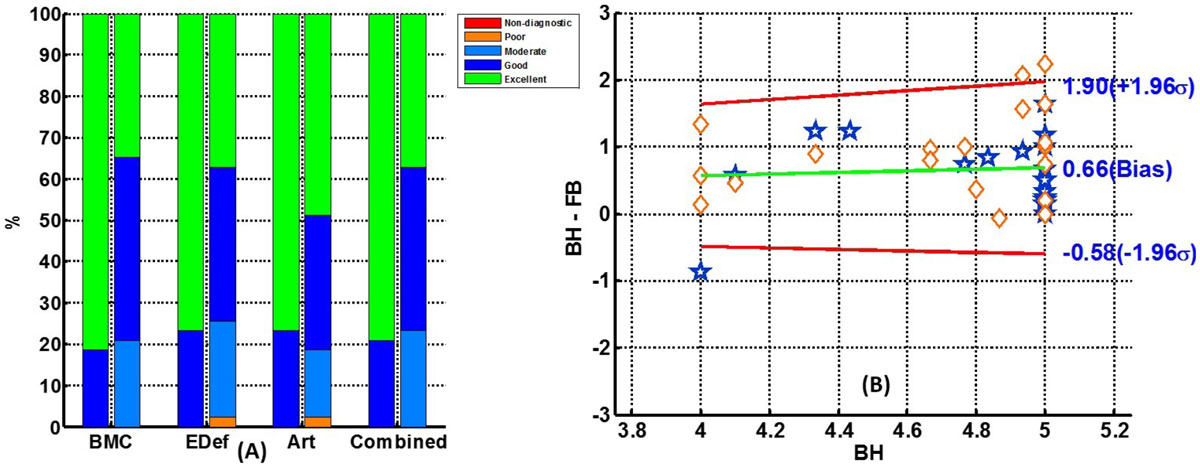
**Bar plot (A) and Bland-Altman plot (B) for clinical scores**. Bar plots depict percentage of patients receiving Excellent, Good, Moderate, Poor clinical scores for specific criterions for blood to myocardial contrast (BMC), endocardial edge definition (Edef), and artifacts (Art). The combined score is the equal weights average of the three scores. Results of the Bland-Altman analyses of clinical score, given by the clinical expert, reveals the change in clinical score for each patient. Horizontal axis of Bland-Altman plot is the clinical score for BH-SSFP and vertical axis is the BH minus RT score. Blue stars correspond to patients from Institute A and orange diamonds those from Institute B.

The RT-SSFP sequence ran successfully in all 43 patients. Total image acquisition time for RT-SSFP (5.9 ± 1.5 min) was significantly longer than conventional BH-SSFP (3.4 ± 1.0 min) (p < 0.001). Combined clinical score was Excellent (80%) to Good (20%) for BH-SSFP and Excellent (38%)-Good (40%)-Moderate (22%) for RT-SSFP (Fig.1). Only in one patient edge definition and motion artifact were scored as Poor.

## Conclusions

While image quality scores were slightly lower for RT-SSFP compared to BH-SSFP, RT-SSFP images were consistently rated as diagnostic quality or better. Previous studies have shown that there was no statistically significant difference in the quantitative metrics of global LV function estimated using RT and BH techniques [1, 2]. We note that a vast majority of subjects in this study were good breathholders (BA plot), and RT-SSFP images were of equivalent image quality. Inclusion of more difficult clinical cases in comparison study may reveal the added value of free breathing cine SSFP alternative. The free breathing RT-SSFP sequence generates diagnostic cine MR images with contrast and spatio-temporal resolutions that are comparable to BH SSFP sequence at the expense of modest sacrifice in image quality in patients able to perform reasonable breath hold. Global LV functional parameters obtained from the two sequences were in good agreement. Thus, FB RT sequence may be a robust alternative for evaluating global LV function in patients with impaired breath-holding capacity.
